# Annotations

**Published:** 1887-11-19

**Authors:** 


### ANNOTATIONS.

**Precocity and Health.**

Perhaps it would be discourteous to inquire too closely into the nature of the enthusiasm which moves the St. James's Hall audiences with regard to Master Josef Hofmann. As a pianist he is remarkable, but nobody pretends that his interpretation of Beethoven is better than Hallé's, or his performance of Chopin superior to Pachmann's. He is in no way greater than these and several other musicians; the interest he excites is aroused by the fact of his being only ten years of age. He is a phenomenon; that is, he is a matter for curiosity—not musical, but pathological. His playing is exceptional for a child of his years, and shows an abnormal mental development. This is certainly a thing to excite attention; but it may be doubted if the attention is wholesome either for audience or performer. The former are roused to an enthusiasm akin to that of the rustic who gazes with open eyes at the bearded lady and the learned pig. The latter is subjected, at a time when quiet and regular habits are essential to the health of both mind and body, to the fatigue of late hours, long and frequent railway journeys, and intense mental excitement. The career of a musician and concert performer is in itself a severe strain on man or woman even of mature age; to a child it is absolutely dangerous. In recent years there have been several instances where child-actors have died from diseases distinctly traceable to the strain of the work on which they have been engaged. Even when less serious consequences result, this premature exhibition of talent is a forcing-frame, which is apt to produce, after unseasonable ripeness, a premature decay of either mind or body. Mozart and Mendelssohn displayed a precocity of talent similar to that of young Hofmann, and both died at an age when most men are at the full maturity of their powers. This is, sad to say, perhaps the best possible ending; it is less disappointing, both to the individual and to the world, than the sterility which often marks the later career of many precocious geniuses—those who promise to take the world by storm at twenty, and leave that promise unfulfilled at fifty. The truth is, that they have exhausted themselves too soon. A man's working life has only a limited duration, and the sooner it begins the sooner it must end. Moreover, if it begins before both mind and body have attained their full stature, the ultimate amount of work done will be inferior both in quantity and quality to what it might have been had the beginning been postponed. *Festina lente* is a good motto for all men in these pushing days; for an artist the adopting of it may make all the difference between temporary and permanent success.

**Colour-blindness in the Mercantile Marine.**

Colour-blindness is a very trivial and often amusing defect in most men, but it is a serious disease in a sailor, and may disqualify him utterly for his work. A man who cannot tell a red from a green light when he is steering a ship may be the cause of a collision with another vessel, in which hundreds of lives may be sacrificed. Therefore the Board of Trade have wisely instituted an examination for colour-blindness which candidates for the posts of master and mate may undergo. But unfortunately it appears from the report recently issued by the Board that the tests adopted for the discovery of colour-blindness are insufficient in themselves, and are applied with considerable laxity. It is true that no fewer than 6.78 per cent. of the candidates are annually rejected on the ground of defective colour vision; which is all the more strange that among the community at large not more than 4 per cent. of the males suffer from this defect, and what makes it more remarkable still is that the examination is not compulsory, and we can easily believe that an unscrupulous seaman who was desirous of becoming an officer would abstain from submitting to it. The inference we draw from this is, that the Board of Trade examiners are unnecessarily severe in their tests, and reject men who would not be looked upon by careful scientists and physicians as suffering from colour-blindness. This bears hardly on individuals, but shows a praiseworthy regard for the public safety on behalf of the Board. While, however, in a grateful spirit we go on reading the report, we find this excess of vigour balanced by a carelessness that is somewhat alarming. Colour-blindness is an incurable disease, yet we find that candidates who have failed to pass the examination are allowed to appear for re-examination, and are frequently passed as free from the defect. This suggests the possibility of any shrewd fellow who will take the trouble to practise the tests upon himself may evade them, and secure the certificate formerly refused to him. Moreover, it appears further that some individuals have been allowed to gain the officer's certificate, and to rise from one grade to another, although they were known to be colour blind. The fact was noted on the back of the certificate, and might therefore to some extent lessen their chances of obtaining an appointment, but the certificate itself was not refused. Again, the rules of the examination seem intended to nullify the chance of satisfactory results. Of the eight or nine colours used as tests some are known as "confusion colours," on account of the greater difficulty the colour blind have in distinguishing them. These tints are the final, the most effective tests; yet it is enacted that a candidate is "not to be regarded as having failed if he miscalls these tints, provided he names the other correctly." Surely it is time that an examination which is at once too severe and too lax should be given up, and a single just and accurate test for colour-blindness provided in its place.

**More Home Claims.**

Our annotation on woollen clothing has excited some comment, and has caused a small invasion of the editorial office. A letter from Dr. Jaeger's representatives in this country will be found elsewhere. We feel, however, that we are doing no injustice if we further point out the value of certain other home manufactures of the woollen kind, which are little better known to the world at large than the Irish woollen goods. We refer to what are called the "Leek" flannels. These are made, not in Ireland, nor in Scotland, nor England, but in that principality of picturesque costumes and incomprehensible speech—Wales. The "Leek" flannels, several examples of which have been sent to us, are made of pure Welsh wool. Dr. Jaeger solemnly disclaims any pretence of having invented a new kind of sheep. Those who are familiar with the qualities of Welsh mutton will be glad to think that the invention of a "new kind of sheep" is as impossible in Wales as in Germany. The Welsh mutton is unsurpassed in excellence, and the wool is as excellent as the mutton. The samples of "Leek" flannel sent to us are beautifully soft in texture and perfect in manufacture—admirably adapted, not only for underclothing, but for all the purposes for which high-class flannels are used. For children's night-gowns these light and warm fabrics are peculiarly suitable. It is not said that no other flannel goods are equal to them; that kind of puffing does not come within the scope of The Hospital's functions. But it is affirmed that these are excellent articles, and the British conductors of The Hospital are always glad to give a helping hand to all kinds of British manufactures.

**A New Fever Hospital.**

The inhabitants of Warwick and Leamington are to be congratulated upon the wisdom displayed by their local rulers in deciding to have a complete and efficient hospital for the treatment of infectious diseases. The new hospital will be erected, from plans prepared by Mr. Keith D. Young, on a site purchased from the Earl of Warwick for £900, the cost of the buildings being estimated at £7,000. Alderman Wackrill, of Leamington, did well to remind his hearers at the laying of the foundation-stone on the 7th inst., that in these days of increasing populations it was necessary, for the protection of great towns and large villages, to resort to isolation to prevent the spread of infectious diseases. The Government did all they could to encourage local authorities to pursue a wise policy in such matters, and they never tired of proclaiming that the object of such hospitals is to protect the public health by removing infectious cases from densely-crowded areas in the earliest stage, and so to secure the health and safety of the families of the citizens. The cost of the hospital and site will be defrayed in this instance out of the loan granted by the Local Government Board, repayable in thirty years. This loan has been raised at 3½ per cent., and will represent a rate of ⅝*d.* in the pound only. We are glad to know that the numbers of infectious hospitals are rapidly increasing throughout the country, and we look forward to the time when it will not be possible to find any district without provision for the isolation and proper treatment of cases of infectious disease.

